# Stability of methods for differential expression analysis of RNA-seq data

**DOI:** 10.1186/s12864-018-5390-6

**Published:** 2019-01-11

**Authors:** Bingqing Lin, Zhen Pang

**Affiliations:** 1 Institute of Statistical Sciences, College of Mathematics and Statistics, Shenzhen University, Shenzhen, China; 2 Department of Applied Mathematics, the Hong Kong Polytechnic University, Hong Kong, China

**Keywords:** Stability, DE analysis, RNA-seq data

## Abstract

**Background:**

As RNA-seq becomes the assay of choice for measuring gene expression levels, differential expression analysis has received extensive attentions of researchers. To date, for the evaluation of DE methods, most attention has been paid on validity. Yet another important aspect of DE methods, stability, is overlooked and has not been studied to the best of our knowledge.

**Results:**

In this study, we empirically show the need of assessing stability of DE methods and propose a stability metric, called Area Under the Correlation curve (AUCOR), that generates the perturbed datasets by a mixture distribution and combines the information of similarities between sets of selected features from these perturbed datasets and the original dataset.

**Conclusion:**

Empirical results support that AUCOR can effectively rank the DE methods in terms of stability for given RNA-seq datasets. In addition, we explore how biological or technical factors from experiments and data analysis affect the stability of DE methods. AUCOR is implemented in the open-source R package AUCOR, with source code freely available at https://github.com/linbingqing/stableDE.

**Electronic supplementary material:**

The online version of this article (10.1186/s12864-018-5390-6) contains supplementary material, which is available to authorized users.

## Background

RNA sequencing (RNA-seq) has now been the most popular technology for genome-wide differential expression (DE) analysis due to its advantages over other technologies, such as high resolution, less bias and relatively low cost. In the past few years, dozens of DE analysis methods have been proposed in three mainstream strategies: (1) Read counts of features are directly fit by a presumed discrete distribution, either Poisson or Negative Binomial (NB) distribution, such as PoissonSeq [1], edgeR [2], DESeq2 [3] and variations of dispersion estimation under both Frequentist and Bayesian frameworks [4, 5]. (2) Raw counts of reads are log-transformed and statistical method based on normal distribution is applied hereafter, like in Voom [6]. (3) No underlying distribution is assumed on read counts, like in SAMseq [7], NOISeq [8] and LFCseq [9]. These methods could avoid possibly misspecified distributions and/or moderate the effect of outliers. While DE methods have been applied to identify features whose expression levels change between conditions and there have been many efforts to systematically compare these methods [10–12], an important question that has not been fully addressed is: how reliable is the selected set of features? Two aspects that are important and of interest to researchers about the reliability of the selected set of features are *stability* and *validity*:

- *Stability* measures the consistency of feature discoveries across datasets from different experiments or platforms. In other words, stability is a metric of reproducibility and answers important questions: if there are small perturbations during the experiments or preprocessing of the datasets, or the experiment was rerun a second time, does the set of selected features remain the same? How similar are these sets of selected features to each other?
- *Validity* measures the similarity between the sets of selected features by DE methods and the true collection of differentially expressed features. In practice, validity is unknown since the true collection of differentially expressed features is unknown. However, some aspects of validity may be estimated, such as false discovery rate (FDR). In simulation studies, one can see a more complete picture of the validity of DE methods by several standard statistical metrics, such as precision, sensitivity, power and receiver operating characteristic (ROC) curves.

The idealized result of DE methods is both high validity and high stability, i.e. sets of selected features are consistent and close to the true set of DE features. Currently, most evaluations of the reliability of DE methods in RNA-seq datasets are focusing on validity [3, 11, 13]. These evaluation procedures ignore the stability of results and may choose DE methods that are highly inconsistent when datasets have small perturbations, i.e. sets of selected features are quite different from each other, but close to the true set of DE features in general.

**Fig. 1:**
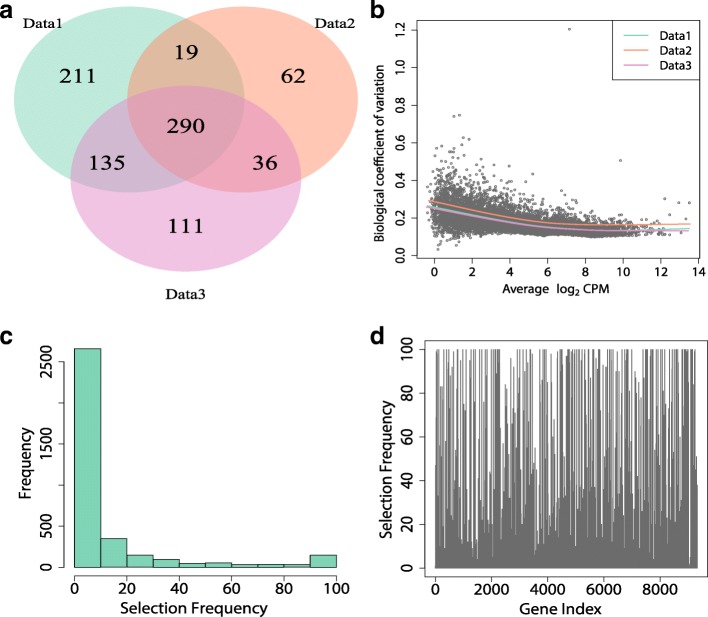
Selection frequency of the Bottomly dataset [23] by edgeR-robust. Bottomly dataset contains ten and eleven replicates of two different, genetically homogeneous mice strains. Sub-datasets are generated by randomly selected five biological replicates for each condition. **a** Venn diagram of 3 randomly selected sub-datasets. **b** Scatterplot of biological coefficient of variation (BCV) against average of log_2_ of counts per million (CPM) of the first randomly selected sub-dataset. Three fitted BCV-CPM trends are represented by different colors. **c** Histogram of selection frequency for 3596 genes that were selected at least once over 100 randomly selected sub-datasets. **d** Selection frequency for each feature over 100 randomly selected sub-datasets

As shown in Fig. 1, DE methods may suffer a lack of stability, i.e. the sets of selected features vary a lot for different subsampled datasets. In particular, although the three randomly generated sub-datasets are similar to each other (Fig. 1b), only 34% features are concordantly selected (Fig. 1a). Furthermore, very few features are consistently selected as DE features over 100 randomly selected sub-datasets (Fig. 1c and d). Particularly, among 3596 features that are selected at least once over the 100 sub-datasets, only 179 features have selection frequency larger than 80 and 2583 features have selection frequency less than 10. Additional file 1: Figure S1 reveals similar findings from the Cheung’s dataset by DESeq2 with 3 replicates for each condition.

**Fig. 2:**
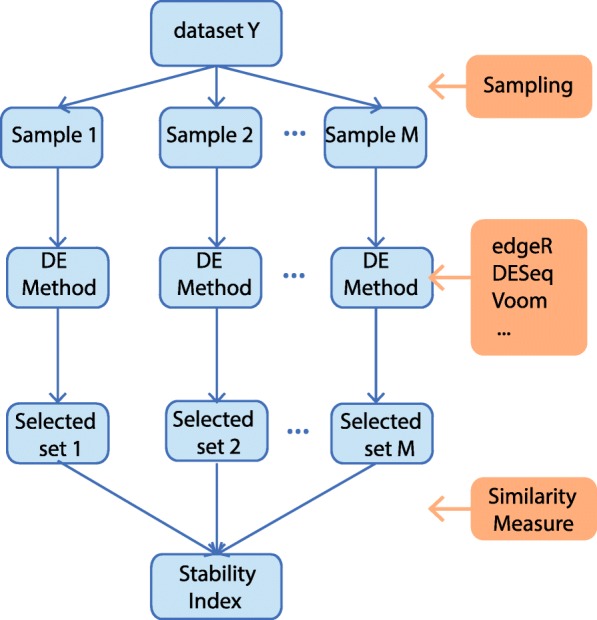
A generic workflow for stability assessment of differential expression analysis. Several perturbed samples are generated from the original dataset by either bootstrap or subsampling in the first step. In the second step, a DE method is applied to each perturbed sample and a subset of features is selected for a given threshold to the *p*-values generated by the DE method. Finally, the stability measure is computed by taking the average of similarities of all pairwise sets of DE features

So far, the major focus of stability measures has been on microarray datasets which have relatively large replicates. Figure 2 depicts a generic workflow for stability assessment of DE methods in microarray datasets that contains three steps: (1) Given a dataset **Y**, *M* perturbed samples are generated by either bootstrap or subsampling; (2) A DE method is applied to each perturbed sample and selects a set of DE features with some given threshold for adjusted *p*-values; (3) The stability measure is computed by taking the average of similarities of all pairwise sets of DE features. Currently, most existing works on stability measures are devoted to developing similarity metrics, including the Jaccard index [14], the consistency index [15], Spearman’s rank correlation coefficient [16], percentage of overlapping genes [17], Pearson’s correlation coefficient [18] and irreproducible discovery rate [19]. As discussed in [18], Pearson’s correlation coefficient is an extension of Jaccard index and Kuncheva’s index [15] and possess many theoretical properties for similarity measure. The proposed metric in this paper, AUCOR, is based on the Pearson’s correlation coefficient.

The above framework suffers from two issues when analysing RNA-seq data, especially when the number of replicates is small. First, in step (1), bootstrapping or subsampling is useless for the typical three-versus-three or five-versus-five cases in RNA-seq datasets, since the number of unique bootstrap or subsampled samples is too limited to be useful. Second, by simply averaging the similarities of pairwise sets of DE features in step (3), the estimates of stability levels may heavily depend on the choice of the size of subsampled samples.

More recently, a new stability metric, called the area under the concordance curve (AUCC), was proposed for single-cell RNA-seq dataset [20]. To calculate the value of AUCC, one ranks the features according to the magnitude of signals in decreasing order, such as *p*-values, then plots the number of features in common among the top *k* features against *k*, for *k*=1,2,…,*K*. The authors adopted the ratio of the area under the curve to the maximal possible value *K*^2^/2 as a measure of concordance. The idea of AUCC is related to the correspondence at the top (CAT) [21] plot. To create a CAT plot, the features are first ranked according to the magnitude of signals in decreasing order as AUCC. For a given list of constants *K*, one plots the proportion of features in common for the top-ranked *K* features against *K*. Both the CAT and the AUCC were developed to measure the similarity of two ranks. Yet, these two metrics can not be used to assess the similarity of two sets of DE features with different sizes. Besides, results of both the CAT and the AUCC depend on the choice of *K*. In [22], the authors defined the measure of stability by the number of common DE features. The idea of this measure is natural and easy to understand. However, if a DE method tends to select large sets of DE features, the size of common features would be large. Yet, similarity metrics more or less have this drawback. From the property of Pearson’s correlation coefficient, we believe that the issue has been alleviated.

The objective of this article is twofold. First, we propose a stability metric to quantify the stability of DE methods based on parametric data perturbations. The idea is to have a sensible measure that can help one decide which DE method should be selected for a RNA-seq dataset at hand in terms of stability. We demonstrate that the proposed metric could well rank the DE methods. Second, we investigate which and how factors of RNA-seq data or DE analysis procedures influence the stability of DE methods in various simulation settings.

## Methods

### Notations

Suppose there are a total of *G* features measured in *n* samples. Let *Y*_*gi*_, *g*=1,…,*G*, *i*=1,…,*n*, be the random variable that expresses the count of reads mapped to the *gth* feature from the *ith* sample and *y*_*gi*_ be the corresponding observed value. The following statistical model is assumed

$$ Y_{gi}\sim \text{NB}\left(\mu_{gi}, \sigma_{gi}^{2}\right) $$

where *μ*_*gi*_ and $\sigma _{gi}^{2}$ are the mean and variance of the Negative Binomial (NB) distribution respectively. In particular, we also assume that feature *g*’s variance equals to $\mu _{gi} + \phi _{g} \cdot \mu _{gi}^{2}$ [4, 13], while the dispersion *ϕ*_*g*_ determines the relationship between the variance $\sigma _{gi}^{2}$ and the mean *μ*_*gi*_.

### Perturbation of NGS datasets

The underlying idea of estimating the stability of DE methods for a specific dataset is simple: If the DE method is stable, then a minor perturbation of the data should not change the set of selected features drastically. Let $f^{gi}_{0}(y)$ be the true density of *Y*_*gi*_, and $f^{gi}_{1}(y)$ be the density of *Y*_*gi*_ with estimated parameters $\hat {\mu }_{gi}$ and $\hat {\sigma }_{gi}^{2}$ respectively. Let *α*_0_ be the probability that a read count is generated from $f^{gi}_{0}(y)$ and *α*_1_=1−*α*_0_ be the probability that a read count is generated from $f^{gi}_{1}(y)$. We generate a perturbed random sample from the mixture distribution

$$ f^{gi}(y) = \alpha_{0} f^{gi}_{0}(y) + \alpha_{1} f^{gi}_{1}(y). $$

Since it is not possible to get the true density of *Y*_*gi*_, $f_{0}^{gi}(y)$, in real datasets, in practice, we generate a perturbed random sample from the mixture distribution as follows.

1. Estimate the mean $\hat {\mu }_{gi}$ and the dispersion $\hat {\sigma }_{gi}^{2}$ for $f_{1}^{gi}(y)$.
2. Generate a random number *p*_*gi*_ that is either 1 or 0 from the Bernoulli distribution with parameter *α*_0_.
3. If *p*_*gi*_=1, set the perturbed observed value from *f*^*gi*^(*y*) as $\tilde {y}_{gi}=y_{gi}$; If *p*_*gi*_=0, set the perturbed observed value from *f*^*gi*^(*y*) as $\tilde {y}_{gi}=y^{*}_{gi}$, where $y^{*}_{gi}$ is generated from the NB distribution with the estimated $\hat {\mu }_{gi}$ and $\hat {\sigma }_{gi}^{2}$.

In other words, we replace the value at location (*g*,*i*) of the dataset by the newly generated number from NB distribution $f_{1}^{gi}(y)$ only if the corresponding generated random number from the Bernoulli distribution is 0. And we keep the value at location (*g*,*i*) of the dataset unchanged if the corresponding generated random number from the Bernoulli distribution is 1. We estimate the dispersions using the procedure proposed by [13] which could sufficiently reduce the effect of outliers and reflect the dispersion and mean trend effectively.

Note that *α*_1_, 0≤*α*_1_≤1, is the perturbation size. If the estimated mean and variance from the original dataset are close to the true mean and variance of the NB distribution, the mixture distribution *f*^*gi*^(*y*) is close to $f^{gi}_{0}(y)$ no matter how we choose *α*_1_. On the other hand, if the estimated mean and variance are not very close to the corresponding true values, the mixture distribution *f*^*gi*^(*y*) can be also close to $f^{gi}_{0}(y)$ when *α*_1_ is small. Due to the small number of replicates in many practical experiments, the mean squared error (MSE) of estimated mean and variance may be large for some features. At each *α*_1_, we generate the perturbed dataset, $\tilde {y}_{gi}$, *g*=1,…,*G*, *i*=1,…,*n*, several times (say M) independently and apply the DE method to each of these perturbed datasets.

### The stability metric of DE methods

The similarity of two sets of selected features, *s*_1_ and *s*_2_, is assessed by the Pearson’s correlation coefficient

$$ \rho(s_{1}, s_{2}) = \text{max}\left(0, \frac{k - k_{1}k_{2}/G}{Gv_{1}v_{2}}\right), $$

where $v_{1} = \sqrt {\frac {k_{1}}{G}\left (1 - \frac {k_{1}}{G}\right)}$, $v_{2} = \sqrt {\frac {k_{2}}{G}\left (1 - \frac {k_{2}}{G}\right)}$, *k* denotes cardinality of the intersection of *s*_1_ and *s*_2_, *k*_1_ and *k*_2_ denote the cardinalities of *s*_1_ and *s*_2_ respectively.

At each perturbation size *α*_1_, compute the average similarities of the new set of selected DE features $s_{m}^{\alpha _{1}}$, *m*=1,…,*M*, and the set of selected DE features *s*_0_ from the original dataset,

$$ \text{Ave}(\alpha_{1}) = \frac{1}{M}\sum\limits_{m=1}^{M} \rho\left(s_{m}^{\alpha_{1}}, s_{0}\right). $$

**Fig. 3:**
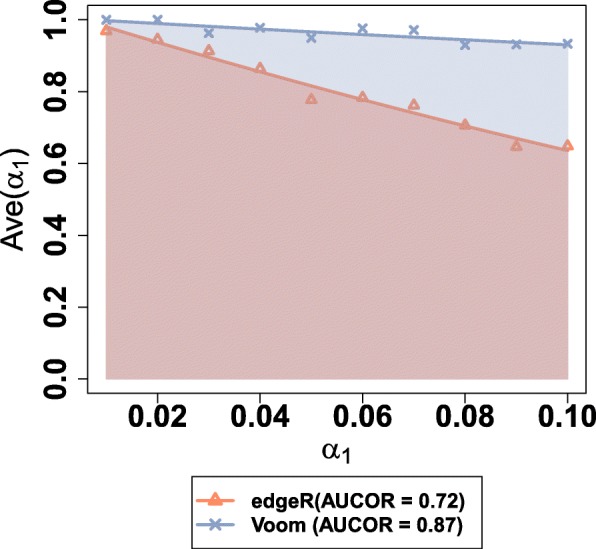
Scatter plot of Ave(*α*_1_) against *α*_1_ for five-versus-five random sub-dataset from Cheung data. *α*_1_ is evenly distributed in (0,0.1). AUCOR is defined as 10 multiplying the area under the Ave(*α*_1_) against *α*_1_ curve

Note that the estimated value of Ave(*α*_1_) depends on the choice of *α*_1_. Ave(*α*_1_) converges to 1 as *α*_1_ tends to 0 and Ave(*α*_1_) shows a decreasing trend as *α*_1_ increases. To alleviate the dependence of the choice of *α*_1_ in the stability metric, we measure the area under the correlation curve that is created by plotting Ave(*α*_1_) at various *α*_1_ from 0 to $\alpha _{1}^{\text {max}}$ (Fig. 3). And finally, the Area Under the Correlation curve (AUCOR) is defined as the area under the correlation curve multiplying $1/\alpha _{1}^{\text {max}}$. We let $\alpha _{1}^{\text {max}}=0.1$ in our numerical experiments to make the dataset generated from the mixture distribution has the similar distribution as the original one (Additional file 1: Figure S2). From empirical experiences, we find AUCOR is not sensitive to the choice of $\alpha _{1}^{\text {max}}$ (Additional file 1: Figure S3).

## Results

### Datasets

To validate the performance of our stability metric, AUCOR, we considered three datasets with relatively large number of replicates for both conditions A and B. This allowed for a split of five vs five or three vs three to mimic the limited number of biological replicates in more generally practical situations. The first, Bottomly [23], compares two genetically homogeneous mice strains, C57BL/6J and DBA/2J. This dataset contains ten and eleven replicates for each condition. The second, Cheung [24], contains read counts for 52,580 Ensemble genes for each of 41 Caucasian individuals of European descent among which there are 17 replicates for female and 24 replicates for male. The third, MontPick [25] from the HapMap project, consists of RNA-seq results from lymphoblastoid cell lines from 129 human samples, among which 60 samples are unrelated Caucasian individuals of European descent (CEU) and 69 samples are unrelated Nigerian Individuals (YRI). For the basic statistics of these three RNA-seq datasets, see Additional file 1: Table S1.

However, the absence of the truth and limited flexibility make the real datasets not suitable to assess the factors that may affect the stability of results of DE analysis. To this end, we also rely on artificial datasets that resemble real datasets as much as possible. We generate datasets from the NB distribution with randomly selected pairs of mean and dispersion computed from Pickrell data [25]. The basic settings are similar to that of [13] as follows. 10,000 features are generated with 6 replicates which are split into two equal-sized groups; 10% of features are simulated as differentially expressed features, among which 50% are set to be up-regulated; fold changes of DE features are generated from the normal distribution *N*(3,0.5^2^). Outliers may also be introduced by multiplying a random factor between 1.5 and 10 to counts of randomly chosen features with probability 0.1.

### DE methods

We consider 7 state-of-art methods for detecting differential feature expression from RNA-seq data, including DESeq [26], DESeq2 [3], edgeR [2], edgeR_robust [13], SAMseq [7], EBSeq [5] and Voom [6]. For version numbers of the softwares and particular parameters used, see Additional file 1: Table S2. We use a common threshold to call a set of DE features. Specifically, DESeq, DESeq2, edgeR, edgeR_robust and Voom all use a threshold of 0.05 for adjusted *p*-values by Benjamini-Hochberg procedure [27]. SAMseq also uses a threshold of 0.05 for the adjusted *p*-values via a permutation-based method, while EBSeq calls DE features with posterior probability of being DE features greater than 0.95.

### Behaviors of AUCOR

We first applied our stability metric, AUCOR, to a 5-versus-5 sub-dataset of Cheung dataset and a simulated dataset. As expected, for all considered DE methods, the similarity metric, Ave(*α*_1_), decreases in general as the increasing of *α*_1_ (Additional file 1: Figure S4 and S5). Compared with the direct use of Ave(*α*_1_) for some specific value of *α*_1_ as the stability metric, AUCOR is a better choice to compare the stability of different DE methods since AUCOR can represent the overall trend of similarities more effectively while the values of Ave(*α*_1_) are a little bit bumpy and the order of DE methods based on Ave(*α*_1_) is not consistent.

To assess the effectiveness of AUCOR, we have to know the true stability level of each DE method, while this is unknown for both real and simulated datasets. Yet, we can find a proxy of the true stability level by computing the average of Pearson’s correlation of DE results for independent samples. Specifically, we treat the real dataset with large number of replicates as population, then independently generate small random samples from this original dataset. For the simulation, we can simply generate multiple random samples from the same NB distribution. In our study, 20 random samples are generated. Then, we apply DE methods to each random sample and compute the Pearson’s correlation coefficients for each pair of random samples. Standard errors of AUCORs are very small relative to the means of AUCOR (Additional file 1: Figure S6), and so these standard errors are not shown in our plots.

**Fig. 4:**
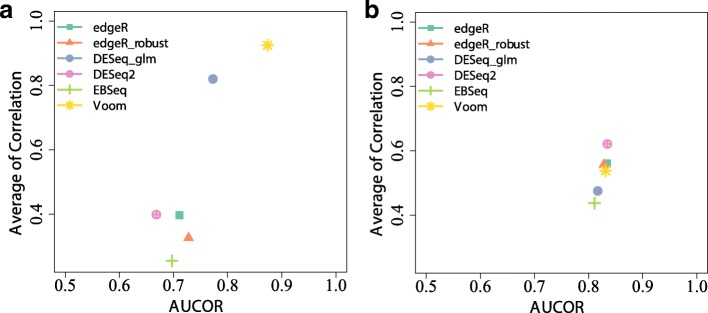
AUCOR against average of correlations among sets of selected features from subsampled datasets. **a** The AUCOR value is computed from a randomly selected 5-versus-5 split of the Cheung data and average of correlations is computed from 20 subsampled 5-versus-5 splits of the Cheung data. **b** Average of correlations is computed from 20 3-versus-3 random simulated samples by using the estimated pair of mean and dispersion of the Pickrell data

The ranking of DE methods for both AUCOR and average of correlation is generally consistent on both real RNA-seq and simulated datasets (Fig. 4, Additional file 1: Figure S7), although the absolute values of AUCOR and averages of correlation coefficients may be distinct a lot. It is noted that the ranks of DE methods for the Cheung dataset and the simulated dataset are quite different. On the Cheung dataset, Voom is most stable, while DESeq2 has relatively low rank. However, on the simulated dataset, DESeq2 is the most stable method. The AUCOR values of SAMseq are zero in these two datasets, because it can hardly produce adjusted *p*-values less than 0.05. Due to the need of large sample size to enable the permutations and the high computational cost, SAMseq is skipped in some comparisons.

**Fig. 5:**
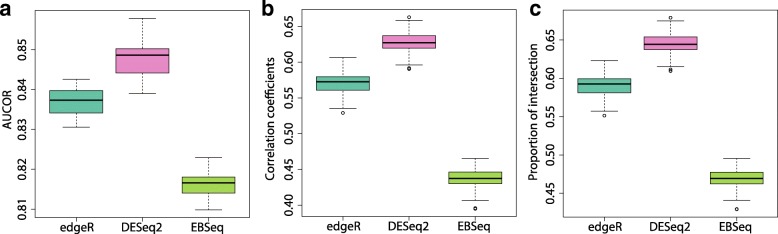
AUCOR, correlation coefficient and proportion of intersection for edgeR, DESeq2 and EBSeq among sets of selected features from simulated datasets using default setting. **a** Boxplot of AUCOR values for 20 experiments. **b** Correlation coefficients of all pairs of 20 datasets generated from same population. **c** Proportion of intersection of all pairs of 20 datasets generated from same population. Proportion of intersection is defined as |*A*∩*B*|/((|*A*|+|*B*|)/2), where *A* and *B* denote two sets of selected features from two different datasets and |*A*| denotes the number of elements in *A*

To further show that the AUCOR values can rank the DE methods according to the stability, Fig. 5 compares stability of edgeR, DESeq2 and EBSeq. All datasets are generated from same population with default setting. Intuitively, stable DE methods select similar sets of features for different datasets. Thus, the correlation coefficient or the proportion of intersection should be large for stable DE methods, and small for unstable DE methods. It is reasonable to treat correlation coefficient or the proportion of intersection as golden standard. From Fig. 5, we can see that the ranking of edgeR, DESeq2 and EBSeq is consistent for AUCOR values, correlation coefficient and the proportion of intersection. In this example, the most stable DE method is DESeq2, followed by edgeR and EBSeq.

**Fig. 6:**
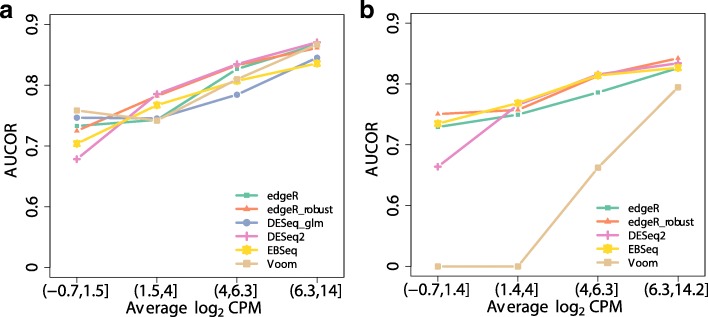
AUCOR values at four abundance levels split by quartiles of average log_2_ CPM: 1.5,4,6.3. The simulated dataset contains 6 replicates evenly split into 2 conditions. **a** AUCOR values at four abundance levels split without outliers. **b** AUCOR values at four abundance levels split with 10% outliers

To understand how the methods perform in the sense of stability with different read count levels, the stability of DE methods is further analyzed. The features in the datasets are separated into four groups by three quartiles of the average of the CPM. All methods exhibit similar patterns for AUCOR values, i.e. it is more stable for the categories of high expressed genes (Fig. 6). Besides, the AUCOR values of all methods are more consistent for the high expressed categories. In the absence of outliers, robust versions of DE methods, such as edgeR_robust and DESeq2, are more stable than other methods, except for the low expressed category. When outliers are introduced, the stabilities of edgeR_robust, DESeq2 and EBSeq only deteriorate slightly, while Voom and DESeq exhibit spectacular drops.

**Fig. 7:**
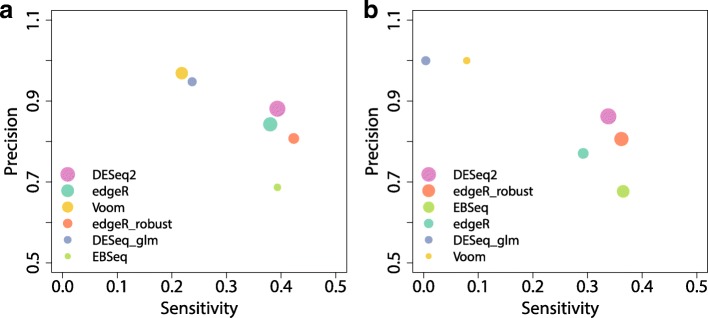
Sensitivity, precision and AUCOR in the simulated dataset. The simulated dataset contains 6 replicates evenly split into 2 conditions. The AUCOR values are represented by the size of points, largest AUCOR values correspond to the largest size of points. **a** Sensitivity, precision and AUCOR in the simulated dataset without outliers. **b** Sensitivity, precision and AUCOR in the simulated dataset with outliers

**Fig. 8:**
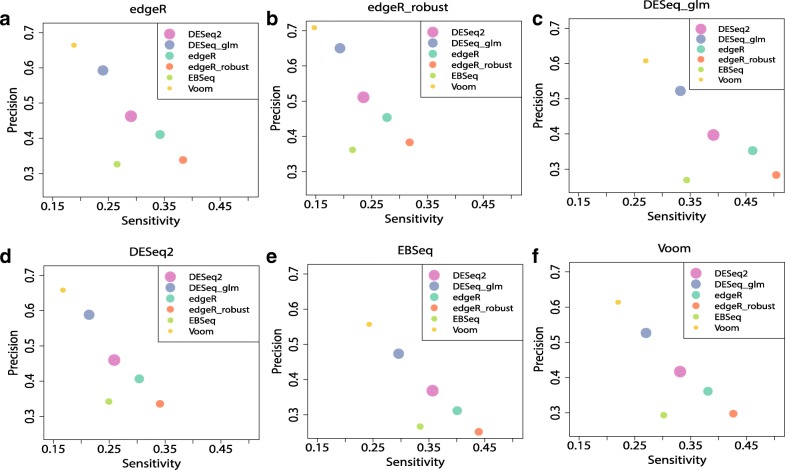
Sensitivity, precision and AUCOR in the 3-versus-3 split of Bottomly dataset. We split Bottomly dataset into a 3-versus-3 evaluation dataset and 7-versus-8 verification dataset. We take the sets of DE genes for the verification dataset by all considered DE methods as truth (we exclude SAMseq, since it could hardly produce adjusted *p*-values less than 0.05). Finally, we have 6 true sets from different DE methods. We then calculate sensitivity and precision values for the results of the evaluation dataset using these 6 true sets in turn. DE method for the verification dataset is labeled on the top of each plot. **a** True set of DE genes from edgeR. **b** True set of DE genes from edgeR_robust. **c** True set of DE genes from DESeq_glm. **d** True set of DE genes from DESeq2. **e** True set of DE genes from EBSeq. **f** True set of DE genes from Voom.

A more comprehensive picture of the performance of different DE methods for the datasets with or without outliers under the basic simulation setting is presented in Fig. 7. The precision-sensitivity curves are provided to assess the validity of the methods, while the size of points represents the level of stability. DESeq2 is clearly the most stable method no matter whether outliers are introduced or not (Fig. 7), while the edgeR_robust and EBSeq also rank at high levels in terms of stability with outliers introduced. When the number of replicates is large, DESeq is the most stable method in the absence of outliers and Voom becomes highly stable even if the outliers are introduced (Additional file 1: Figure S9). DESeq2, edgeR and edgeR_robust have relatively high sensitivity. Their sensitivity values are around 0.4 which seems satisfactory in such small sample cases. In terms of precision, Voom and DESeq perform better than other methods (Fig. 8 and Additional file 1: Figure S10). Precision values of both methods can be around the nominal level 0.95. Similar findings are observed for datasets with outliers, although both sensitivity and precision are slightly worse.

### Factors that affect stability of DE results

While AUCOR is useful to verify how well DE methods behave in terms of stability for a dataset at hand, and from which a method having high stability can be chosen, it is also of interest to investigate which and how underlying factors affect the stability of DE analysis results. we consider some potential factors and their corresponding levels as follows:

1. nSamp: sample size varies from 2 to 50, the default is 3.
2. gFeatures: number of features varies from 2000 to 20,000, the default is 10000.
3. pDE: percentage of differentially expressed features varies from 10% to 70%, the default is 10%.
4. mFoldChange: mean of fold change of DE features, varies from 3 to 6, the default is 3.
5. rDisp: ratio that is multiplied to the estimated dispersion of the original dataset, varies from 0.6 to 2, the default is 1.
6. pUp: proportion of DE features that are up-regulated, varies from 0.1 to 0.7, the default is 0.5.
7. threshold: cutoff point to adjusted *p*-values which are 0.001,0.01,0.05,0.1 and 0.2, the default is 0.05.
8. pOutlier: proportion of outliers, varies from 0.1 to 0.5, the default is no outlier.
9. outlierMech: three mechanisms that are used to generate outliers: S, R and M [13]. Random factors are generated from a Uniform distribution *U*(1.5,10). In mechanism S, features are randomly selected with some probability and one read count among samples of each selected feature is multiplied by a random factor. In mechanism R, each read count in the dataset is selected with some probability to be multiplied by a random factor. In mechanism M, each read count in the dataset is selected with some probability, and if so, the selected read count is resampled by a NB distribution with mean *μ* multiplied by a random factor. In mechanism S, each feature has at most one outlier, while in mechanism R and M, features may have more than one outliers. The default is no outlier.

#### Impact of number of replicates on stability

**Fig. 9:**
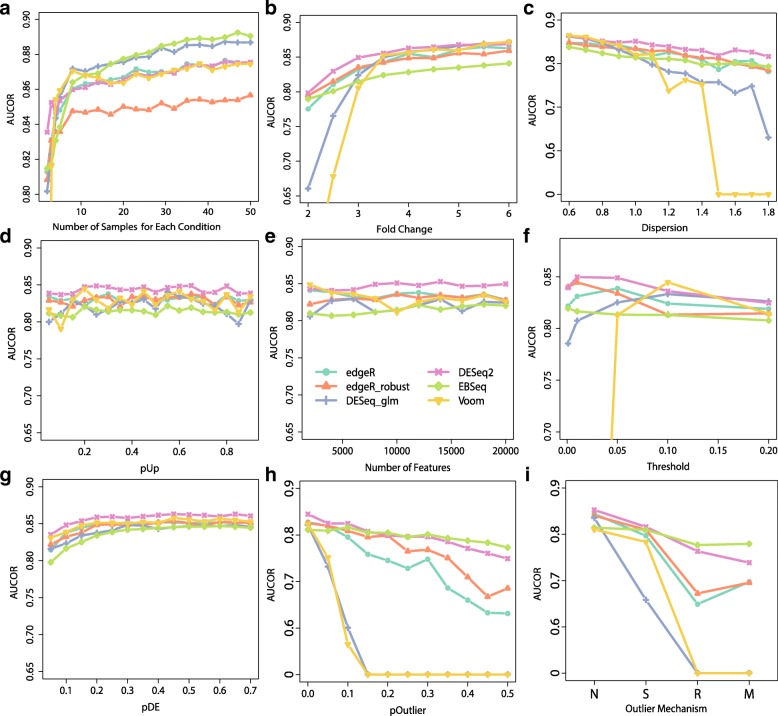
Impact of factors on stability. **a** AUCOR against number of samples for each condition that are 2,3,4,5,8,11,14,…. **b** AUCOR against fold change. Fold changes of DE features are generated from the normal distribution with standard error 0.5 and the means of fold changes are set as 2,2.5,3,…,6. **c** AUCOR against dispersion. Basic pairs of mean and dispersion are randomly selected from that of Pickrell data [25]^16^. Dispersions are adjusted by multiplying a ratio from 0.6 to 2 with step size 0.1. **d** AUCOR against proportion of DE features that are up-regulated. **e** AUCOR against number of features. **f** AUCOR against threshold. Features with adjusted *p*-values less than the threshold are identified as DE features. We consider 5 commonly used thresholds: 0.001,0.01,0.05,0.1,0.2. **g** AUCOR against proportion of DE features that is spread from 10% to 70%. **h** AUCOR against proportion of outliers. **i** AUCOR against outlier mechanisms: N, S, R and M. N represents the case without outliers. Different DE methods are represented by different symbols and colors

**Fig. 10:**
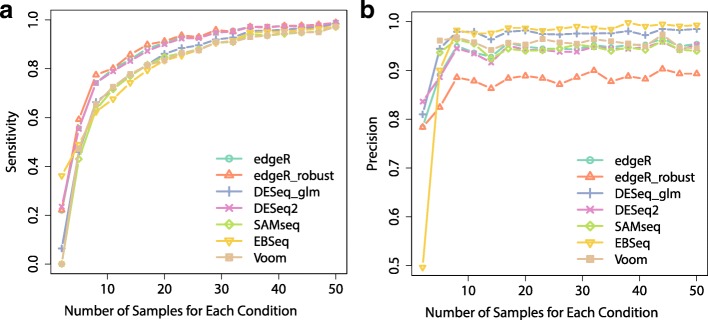
Sensitivity and precision against number of samples for each condition. **a** Sensitivity curves. **b** Precision curves. Simulated factors except number of samples are held as the basic setting. Different DE methods are represented by different colors

Among the 9 potential factors listed above, number of replicates may be the one that researchers can control easily. So, we are particularly interested in the performance of DE methods on the RNA-seq dataset as the increasing of number of replicates. As expected, AUCOR values of all methods increase as the number of replicate increases (Fig. 9a). In particular, we note that the AUCOR values experience a two-phase process, a sharp increase as the number of replicates is less than 10 for each condition followed by a slight increase as the number of replicates is above 10. When the number of replicates is 2 for each condition, DESeq2 is the most stable method, followed by edgeR, edgeR_robust, EBSeq, and DESeq, while Voom is highly unstable. However, when the number of replicates reaches 8, Voom and EBSeq are the most stable methods, followed by edgeR, edgeR_robust and DESeq2, while the edgeR_robust become the least stable method. We also observe that the precisions of these DE methods have similar patterns as AUCOR and ranks of methods according to AUCOR and precision are overall consistent (Figs. 9a and 10). It is also interesting, as a byproduct, to see that both AUCOR and precision can barely increase as the number of replicates reaches some point (in our example, the change point is around 10), while the sensitivity can continuously increase and tends to 1 when the number of replicates is sufficiently large.

#### Impact of fold change and dispersion on stability

Fold change and dispersion are two important factors that may affect the stability of DE methods, since these two factors are the main parameters that all DE methods directly or indirectly want to estimate, and results of DE methods are largely determined by the qualities of the estimates of fold change and dispersion. Intuitively, as the increasing of fold change, the difference between DE features and non-DE features are larger, and as a result, it is easier for DE methods to identify DE features. By contrast, as the increasing of dispersion, the difference between DE features and non-DE features becomes vaguer, and it is more difficult to find DE features for DE methods. In general, as the increasing of fold change or decreasing of dispersion, all DE methods exhibit higher stability (Fig. 9, Additional file 1: Figures S11(a) and S11(b)). When the number of replicates is 3 for each condition, DESeq and Voom decrease sharply when the fold change is small or dispersion is large. When the number of replicates is 10 for each condition, DESeq and Voom show high stability and the stability trends of these two methods are similar to that of other DE methods (Additional file 1: Figures S11(a) and S11(b)).

#### Impact of outliers on stability

As shown in [3, 7, 13], outliers may appear in RNA-seq by various reasons, such as GC content and specific characteristic of individuals. And the presence of outliers may influence the estimates of parameters of DE methods and consequently the finally calling of DE genes. As the increasing of proportion of outliers, Voom and edgeR can not identify any DE genes when the proportion of outliers is larger than 15%, while AUCOR values of EBSeq and DESeq2 only decrease slightly (Fig. 9h, Additional file 1: Figures S12(c) and S12(d)). Regarding to the outlier generating mechanism, we can also observe the similar pattern, i.e. DESeq2 and EBSeq achieve highest AUCOR values no matter which outlier mechanism is adopted.

#### Impact of number of features, pDE, pUp and threshold on stability

Threshold is another factor that one can control. Figure 9f shows that the stability of DE methods may be largely affected by different choices of threshold. We can see that the number of features and the proportion of up-regulated features also do not influence the stability (Fig. 9d and e). The proportion of DE features influences the stability slightly. DE methods seem less stable when the proportion of DE features is small (Fig. 9g). And the patterns for all methods are consistent.

## Discussion

As RNA-seq has become the assay of choice for high-throughput gene expression analysis, differential expression analysis for RNA-seq dataset has received extensive attention of researchers and practitioners. The main goal of DE analysis is to find a set of features toward a task such as classification or identification of the top relevant features corresponding to a biological phenomenon of interest. Regarding to the reliability of DE methods, there are two essential aspects: stability and validity. To date, most attention has been paid on validity, while stability is overlooked during the evaluation of DE methods. Thus, the current evaluation system for DE methods may prefer methods with low reproducibility.

We have used three different datasets with large number of replicates, Bottomly, Cheung and PickMont datasets, to illustrate the stability of the DE methods. We observed that the selected sets of features were highly variable for different randomly sampled sub-datasets. This demonstrated the need for assessing stability and prompted us to propose a stability metric AUCOR, which generates the perturbed datasets by a mixture distribution and combines the information of similarities between the sets from perturbed datasets and the original dataset by the area under the correlation curve which could effectively alleviate the influence of the choice of perturbed size on the stability metric. We empirically demonstrated the effectiveness of AUCOR by showing the consistency of ranks of DE methods according to the AUCOR and averages of correlations from subsampling (Fig. 4).

An advantage of the proposed stability metric is the suitability to RNA-seq datasets with small number of replicates under both conditions. This advantage is critical, since the number of replicates is still small in many RNA-seq studies due to the limited budget, precious samples or rare cell types in some cases. This property of the proposed stability metric relies on a key assumption: read count follows a NB distribution whose parameters are properly estimated. First, the NB distribution assumption is widely used in quantifying expression levels of RNA-seq datasets and generally a reasonable assumption for read counts [5, 11, 22]. We estimate the dispersions using the procedure proposed by [13] which could sufficiently reduce the effect of outliers and reflect the dispersion and mean trend effectively. Second, we set the maximum size of perturbation as 0.1 which further dampens the effect of possibly violation of assumption or invalid estimates of parameters. The overall trends of mean and dispersion for the perturbed datasets are very close to those of the original datasets (Additional file 1: Figure S2).

In this study, we further employed simulations to explore which and how underlying factors affect the stability of DE analyses via a broad range of possible settings. Our findings can be summarized as follows. First, levels of fold change of truly differentially expressed features and dispersions of the dataset substantially affect the stability of DE methods. Specifically, as the decreasing of fold change or increasing of dispersion, DE methods tend to be less stable. Second, as expected, more replicates could make the results of DE methods more stable. However, the stability of all methods only increases slightly after the number of replicates reaches some value, in our example, 10. Third, outliers also reduce the stability as well as validity. Fortunately, anti-outlier schemas used by either DESeq2 or edge_robust can successfully alleviate the influences of outliers and make the AUCOR values decrease slower.

Further, it is worth mentioning that the perturbation of dataset is based on the assumption of the NB distribution. Although in most cases NB distribution is a proper assumption and the value of $\alpha _{1}^{\text {max}}$ is restricted to a small scale to avoid the possible violation of the NB distribution or poor estimation of parameters, complete violation of the assumption can possibly lead to undesired results. A nonparametric method for perturbation will be required to solve this problem. We leave this to the future work.

## Conclusion

In conclusion, we developed a metric to measure the stability of DE methods for differential expression analyses of RNA-seq data. Overall, the metric could rank DE methods according to the stability levels. There is no single DE method which can be most stable in all cases. On one hand, we summarize stability performance of 6 popular DE methods based on our study (Table 1). The practitioners can choose a method according to the table based on the information of the given RNA-seq dataset. On the other hand, practitioners can choose some valid candidate methods for the specific data based on the evidence of extensive numerical comparisons and theoretical backing in the literature, then estimate the stability levels of these candidate DE methods by AUCOR and select a DE method according to AUCOR values.

**Table 1:** Summary of stability levels based on AUCOR

	edgeR	edgeR_robust	DESeq_glm	DESeq2	EBSeq	Voom
Low replicate number	+	+	+	++	-	–
(2 to 4)						
High replicate number	+	-	++	+	+	++
(> 4)						
Low fold change	+	+	–	+	+	–
(< 3)						
High fold change	+	+	+	+	-	+
(> 3)						
Low dispersion	+	+	-	+	+	–
(< 1)						
High dispersion	+	+	+	+	+	+
(> 1)						
No outliers	+	+	+	+	+	+
Outliers	-	+	–	++	++	–
Low expressed features	+	+	+	-	-	+
High expressed features	+	+	-	+	-	+

Symbols, ++, +, −, −− indicate very good, good, bad and very bad, respectively

In this paper, we focus on assessing the stability of selected sets of DE features based on a pre-set threshold for the ranking of features from DE methods. Thus, this stability metric depends on the choice of the threshold and may have some potential drawbacks. First, features whose *p*-values are close to the pre-set threshold on both sides will be treated very differently. This may potentially affect the stability level of DE methods, although in general this is not a big issue. Usually there are not many features’ adjusted *p*-values close to the threshold. If it does happen, this may indicate that the DE method is not able to provide stable results since small perturbation of the dataset may result in very different collection of features. Second, the proposed approach measures the stability of selected subsets of features, but not the ranking of features by DE methods. The information from interior rankings in selected subsets is overlooked. We believe that the proposed method can be readily extended to consider similarity of the weight values of features (such as *p*-values) or the ranking of features. Besides, there are other similarity measures for the results of DE methods other than Pearson’s correlation coefficient. It is also of interest to fully study how other similarity measures can be incorporated into our framework. We will leave this as the future work.

## Additional file

Additional file 1 Supplementary text and figures. This file contains related codes to use existing approaches, information and results for simulated and real datasets. (PDF 953 kb)

## Abbreviations

AUCOR: Area under the correlation curve
DE: Differential expression
NB: Negative binomial distribution
ROC: Receiver operating characteristic
