# Supplementary material for: Stability of methods for differential expression analysis of RNA-seq data
Source: BMC Genomics. 2019 Jan 11;20:35. doi: 10.1186/s12864-018-5390-6 (PMC6330476; doi:10.1186/s12864-018-5390-6)
Supplement: Supplementary file 1 — Supplementary text and figures. This file contains related codes to use existing approaches, information and results for simulated and real datasets. (PDF 953 kb) [file 12864_2018_5390_MOESM1_ESM.pdf]

Supplementary Materials to "Stability of methods for differential expression analysis of RNA-Seq data "

Bingqing Lin[1], Zhen Pang[2]

[1]Institute of Statistical Sciences, College of Mathematics and Statistics, Shenzhen University, Shenzhen 518060

China

[2] Department of Applied Mathematics, the Hong Kong Polytechnic University

Hong Kong

1 Supplementary Tables

Table S1: Basic statistics of three RNA-seq datasets and numbers of differentially expressed genes for seven DE methods with a threshold 0.05 for adjusted P- values. All three datasets are downloaded from ReCount (Frazee et al., 2011). We can see that PickMont apparently has more significant genes than other two datasets. On the other hand, Cheung only has around ten significant genes.

Dataset	Condition A	Condition B	# of Genes	# of Genes after filtered	Source
Bottomly	10 C57BL/6J	11 DBA/2J	36536	9323	Bottomly et al. (2011)
Cheung	17 Female	24 Male	52580	7145	Cheung et al. (2010)
PickMont	60 CEU	69 YRI	52580	7104	Pickrell et al. (2010) and Montgomery et al. (2010)

Dataset	DESeq	DESeq2	edgeR	edgeR_robust	SAMseq	EBSeq	Voom
Bottomly	1044	1321	647	1170	0	495	901
Cheung	11	18	5	9	0	24	6
PickMont	4345	4687	3271	4354	0	2933	4284

Table S2: Versions of packages used in the article.

package	version	additional information
edgeR	3.16.5	edgeR standard pipeline
edgeR_robust	3.16.5	edgeR-robsut pipeline
DESeq	1.26.0	use the GLM test
DESeq2	1.14.1	DESeq2 standard pipeline
SAMseq	2.0	SAMSeq standard pipeline
EBSeq	1.14.0	EBSeq standard pipeline
Voom	3.30.8	limma voom standard pipeline

2 Supplementary Figures

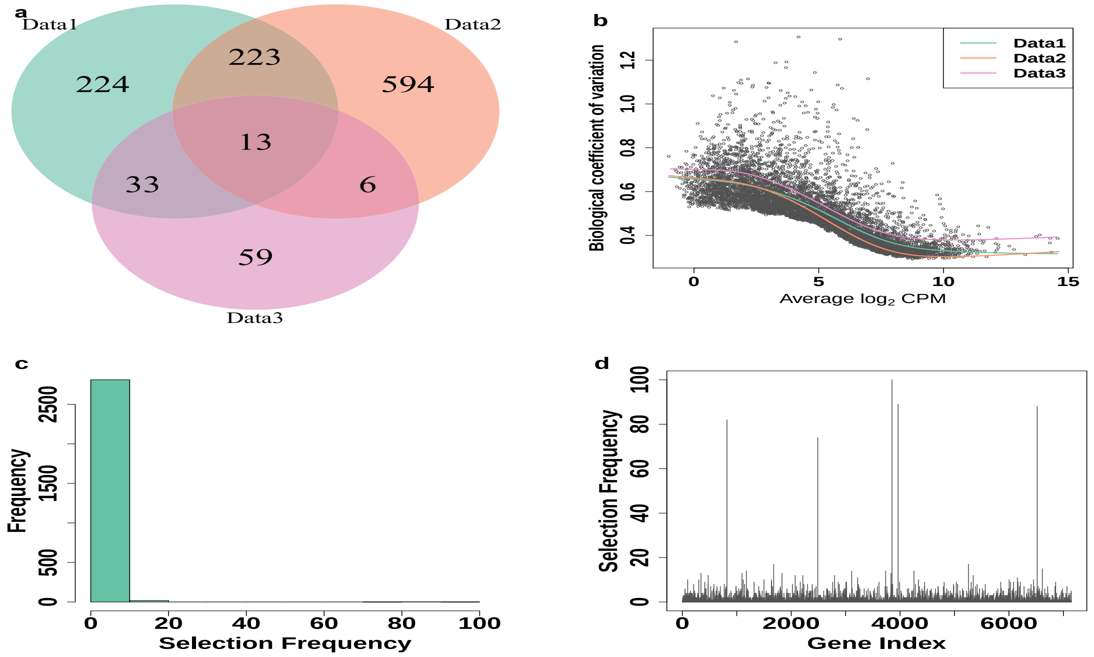

Figure S1: Selection frequency of the Cheung' RNA-seq dataset (Cheung et al., 2010) by DESeq2. Cheung's dataset contains RNA-seq count data from lymphoblastoid cell lines from 17 and 24 unrelated Caucassian Female and Male individuals of European decent, respectively. Sub-datasets are generated by randomly selected three biological replicates for each condition. (a) Venn diagram of 3 randomly selected sub-datasets . (b) Scatterplot of BCV against CPM of the first randomly selected sub-dataset. Three fitted BCV-CPM trends are represented by different colors. (c) Histgram of selection frequency for 4580 genes that were selected at least once over 100 randomly selected sub-datasets. (d) Selection frequency for each gene over 100 randomly selected sub-datasets.

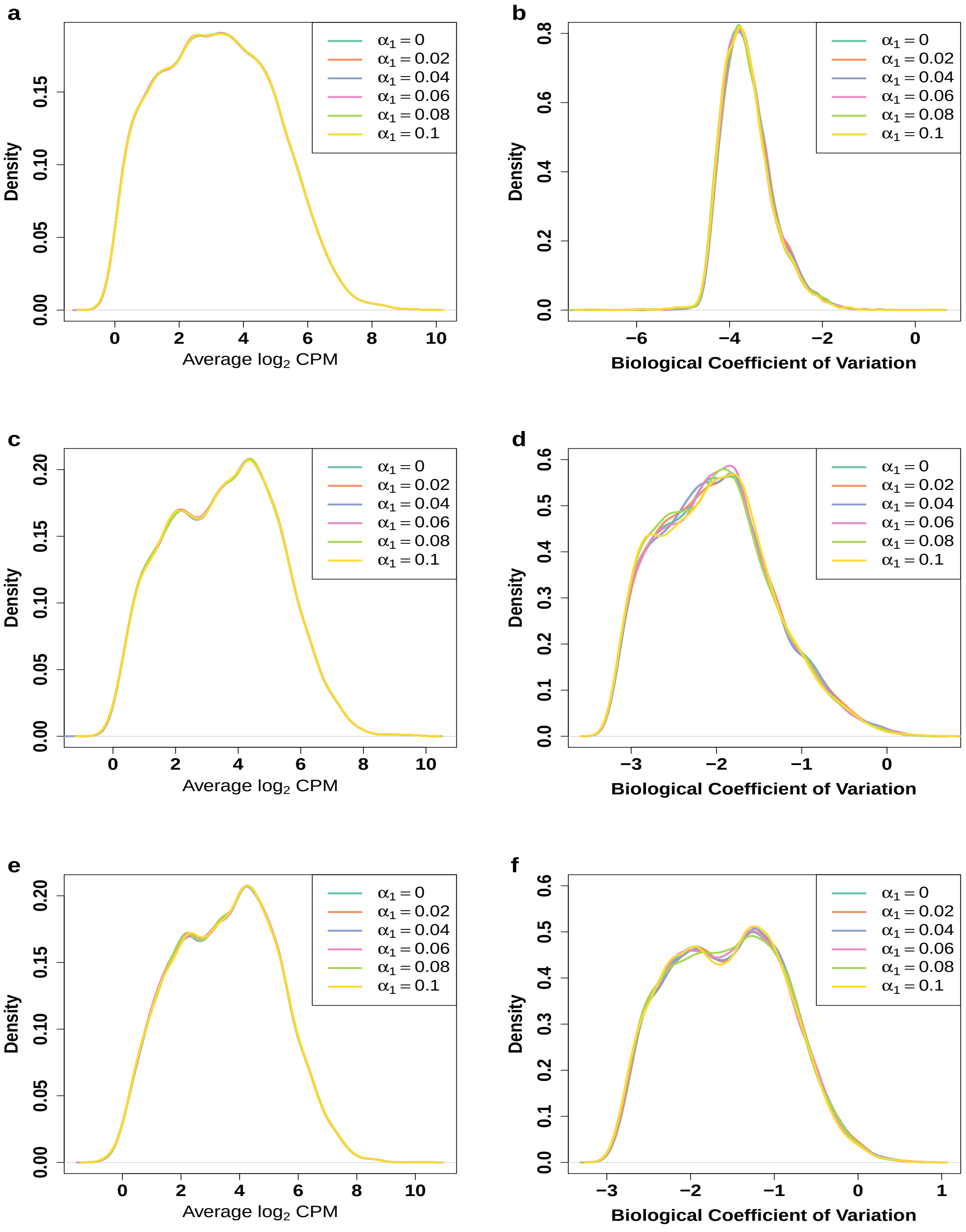

Figure S2: Distributions of means and dispersions of datasets generated from mixture distribution. The RNA-seq datasets are five-versus-five sub-datasets randomly selected from Bottomly, Cheung and PickMont datasets. The left panels show the distributions of means for varying α_1. The right panels show the distributions of dispersions. Averages of correlations for pairs of replicates between original datasets and the generated datasets with $\alpha_1 = 0.1$ for three datasets are 0.995, 0.990 and 0.994, respectively. (a)-(b) Bottomly datasets. (c)-(d) Cheung datasets. (e)-(f) PickMont datasets.

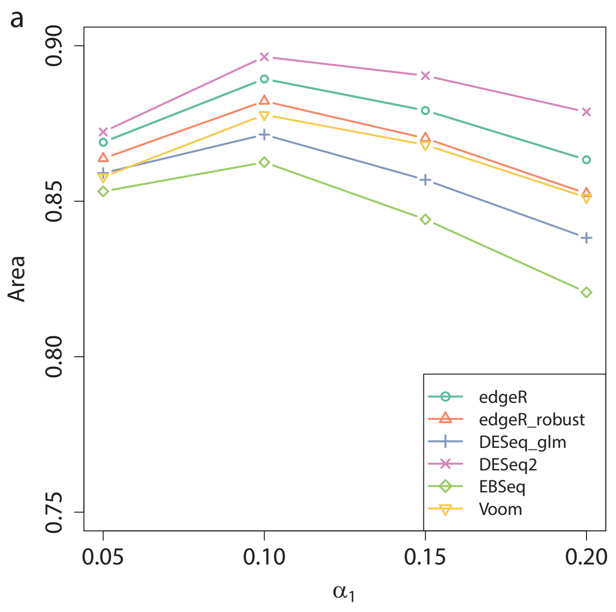

Figure S3: Area under correlation curve for varying $\alpha_1^{\max}$ that is set as 0.05, 0.1, 0.15 and 0.2. (a) The dataset is generated using the basic setting. (b) Bottomly dataset is randomly split into a 3-versus-3 dataset.

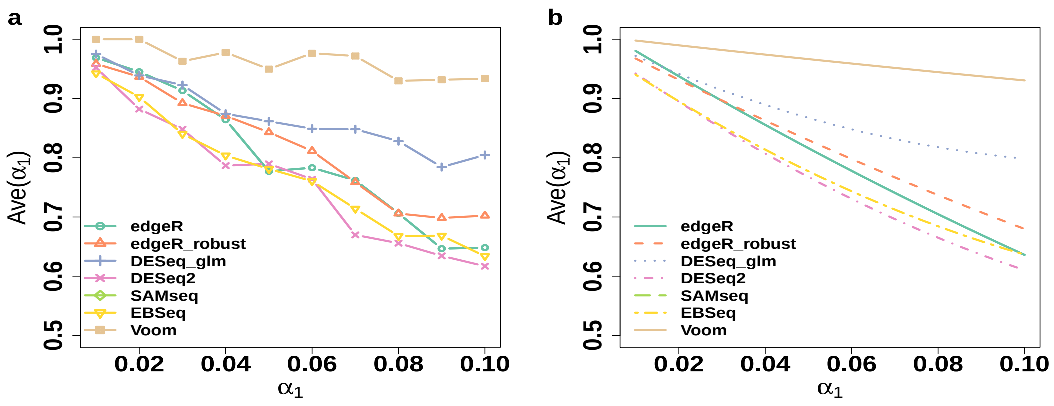

Figure S4: The overall trend of the average of similarities against the proportion of perturbed read counts for the randomly selected 5 versus 5 split of the Cheung data (Cheung et al., 2010). (a) Scatter plot of Ave(α_1) against α_1. Each line corresponds to one of the DE methods. Different DE methods are represented by different symbols and colors. α_1 is evenly distributed in $(0, 0.1)$. (b) Fitted lines of Ave(α_1) against α_1. As expected, the average of similarities decreases as the proportion of perturbed read counts increases.

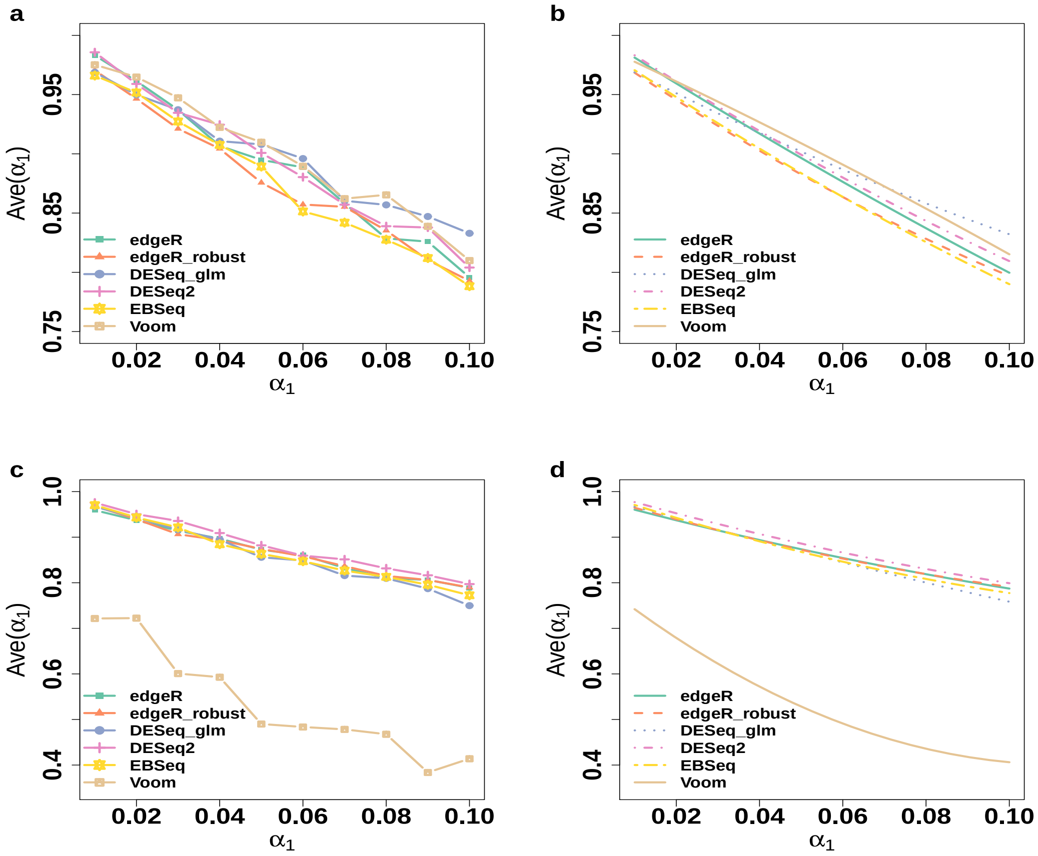

Figure S5: The overall trend of the average of similarities against the proportion of perturbed read counts for the randomly selected three-versus-three and five-versus-five splits of the Bottomly and PickMont datasets. (a) and (c) Scatter plot of Ave(α_1) against α_1 for Bottomly and PickMont dataset. Each line corresponds to one of the DE method. Different DE methods are represented by different symbols and colors. α_1 is evenly distributed in $(0, 0.1)$. (b) and (d) Fitted lines of Ave(α_1) over α_1 for Bottomly and PickMont dataset. As expected, the average of similarities decreases as the proportion of perturbed read counts increases.

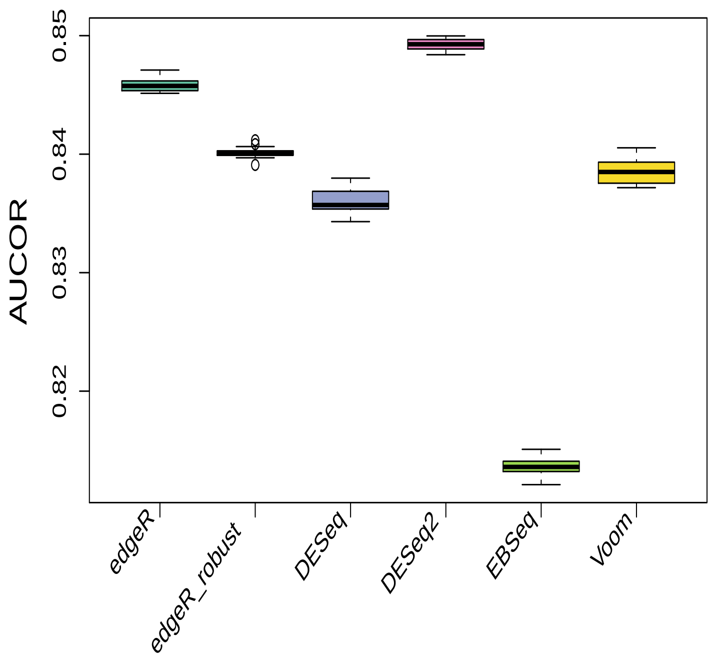

Figure S6: Boxplots of AUCORs of different DE methods for the basic simulated setting. The AUCORs for each method is calculated 20 times.

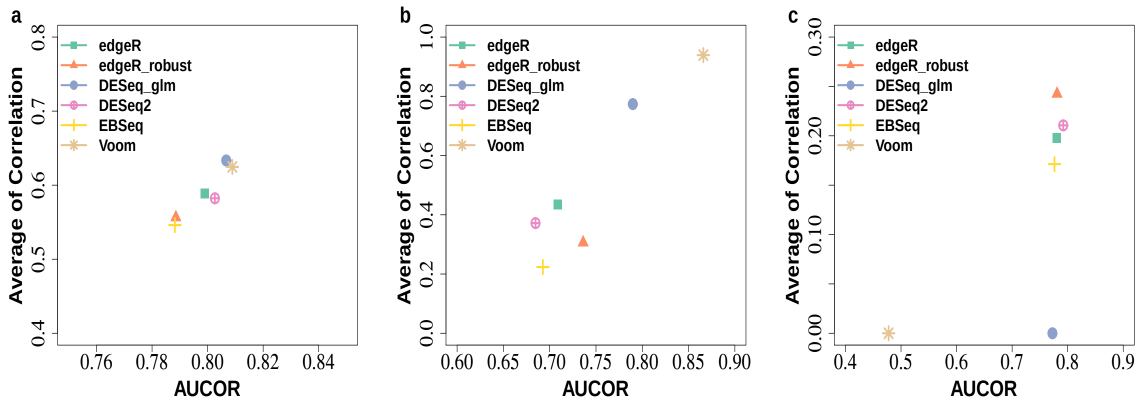

Figure S7: AUCOR against average of correlations among sets of selected features from sub-sampled datasets. (a) Three-versus-three split of Bottomly dataset. (b) Five-versus-five split of Cheung dataset. (c) Five-versus-five split of PickMont dataset. Ranks according to AUCOR and averages of correlations are generally consistent for all three datasets.

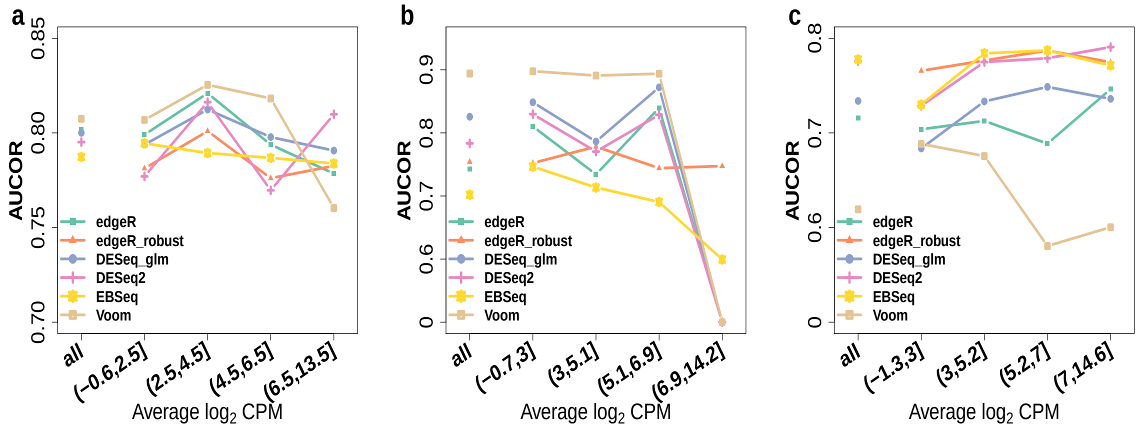

Figure S8: AUCOR values at four abundance levels split by quartiles for Bottomly, Cheung and PickMont datasets . (a) AUCOR values at four abundance levels from 3-versus-3 split of Bottomly dataset. (b) AUCOR values at four abundance levels from 5-versus-5 split of Cheung dataset. (c) AUCOR values at four abundance levels from 5-versus-5 split of PickMont dataset.

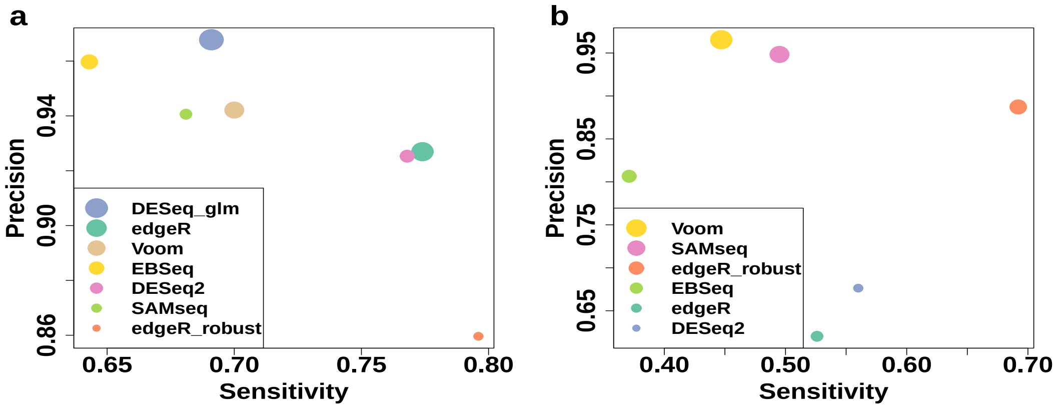

Figure S9: Sensitivity, precision and AUCOR in the simulated dataset when there are 10 replicates for each condition. The AUCOR values are represented by the size of points, largest AUCOR values correspond to the largest size of points. (a) Sensitivity, precision and AUCOR without outliers. edgeR_robust achieves highest sensitivity score, but lowest precision. DESeq are edgeR are two most stable methods in the absence of outliers. DESeq has very high precision score, but relatively low sensitivity score. By contrast, edgeR has very low precision score, but relatively high sensitivity score. (b) Sensitivity, precision and AUCOR with outliers. With outliers, Voom and SAMseq become the most stable methods when number of replicates are relatively large. edgeR_robust remains high sensitivity to identify DE features.

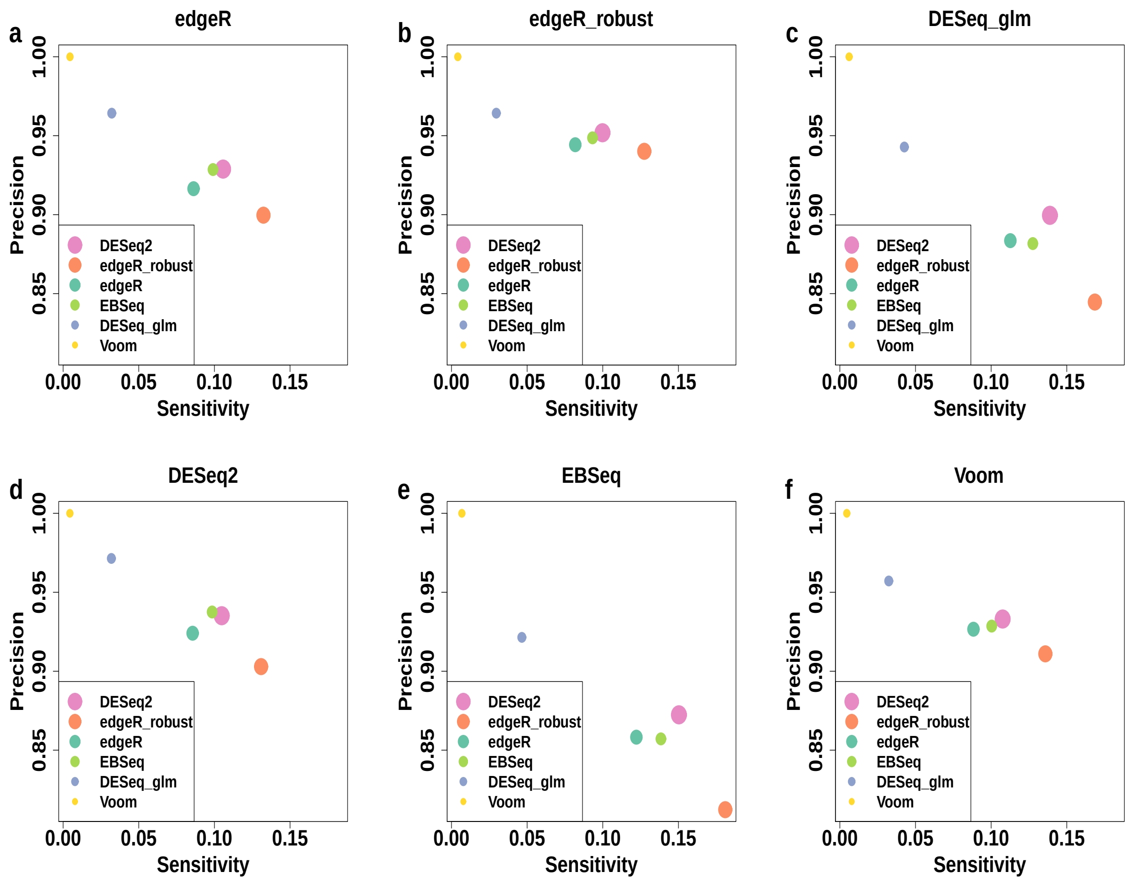

Figure S10: Sensitivity, precision and AUCOR in the 5-versus-5 split of PickMont dataset. We split PickMont dataset into a 5-versus-5 evaluation dataset and 55-versus-60 verification dataset. We take the sets of DE genes for the verification dataset by all DE methods considered (we exclude SAMseq, since it could hardly produce adjusted P-values less than 0.05.) as truth. Finally, we have 6 true sets from different DE methods. We then calculate sensitivity and precision values for the results of the evaluation dataset using these 6 true sets in turn. DE method for the verification dataset is labeled on the top of each plot. all plots show similar patterns no matter which method is chosen to call DE genes in the verification dataset. DESeq2 has comparable sensitivity and precision values to edgeR and EBSeq, but more stable than these two methods. edgeR_robust has relatively high sensitivity, but low precision in general. By contrast, Voom is too conservative and results in very low sensitivity but high precision.

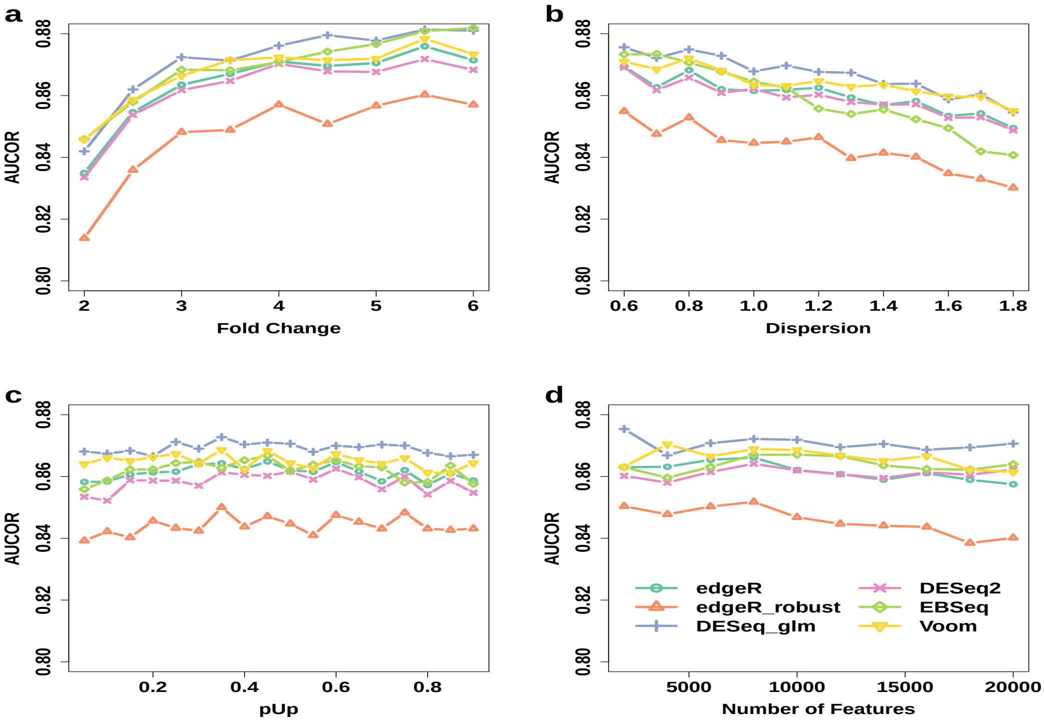

Figure S11: Impact of factors on stability when there are 10 number of replicates for each condition. (a) AUCOR against fold change. Fold changes of DE features are generated from normal distribution with standard error 0.5 and the mean of fold changes are set as $2, 2.5, 3, \ldots, 6$. (b) AUCOR against dispersion. Basic pairs of mean and dispersion are randomly selected from that of Pickrell data (Pickrell et al., 2010). Dispersions are adjusted by multiplying a ratio from 0.6 to 2 with step size 0.1. (c) AUCOR against proportion of DE features that are up-regulated. (d) AUCOR against number of features. Different DE methods are represented by different symbols and colours. These four factors have similar influences on all methods. As the increasing of fold change or decreasing of dispersion, all methods are more stable. pUp and number of features do not have significant influences on the stability of all methods. DESeq and Voom are generally the most stable methods in all situations in this simulated setting.

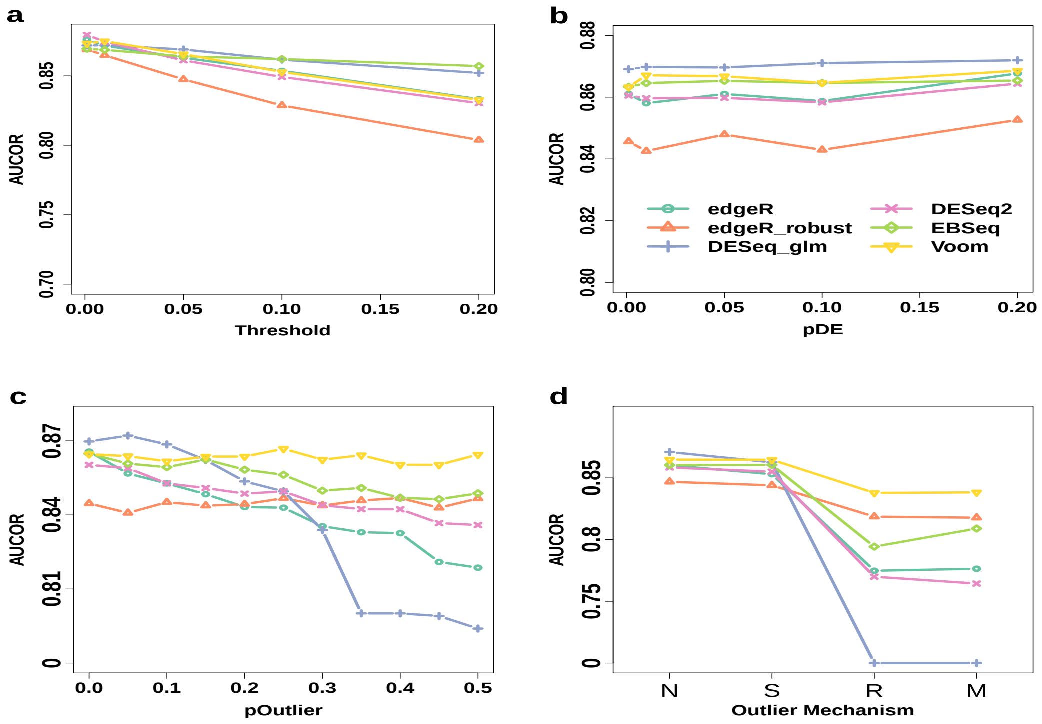

Figure S12: Impact of factors on stability when there are 10 number of replicates for each condition. (a) AUCOR against threshold. Featurs with adjusted P-values less than the threshold are identified as DE features. We consider 5 often used thresholds: $0.001, 0.01, 0.05, 0.1, 0.2$. (b) AUCOR against porportion of DE features that is spread from 10% to 70%. (c) AUCOR against proportion of outliers. (d) AUCOR against outlier mechanisms: N, S, R and M. N represents the case without outliers. Different DE methods are represented by different symbols and colours. Threshold and proportion of DE features do not have significant influences on the methods. When outliers are introduced, DESeq deteriorates a lot, while Voom remains the most stable method.

References

Bottomly, D., Walter, N., Darakjian, J. H. P., Kawane, S., buck RP Searles, K., Mooney, M., McWeeney, S., and Hitzemann, R. (2011). Evaluating gene expression in c57bl/6j and dba/2j mouse striatum using rna-seq and microarray. *PloS ONE*, 6:17820.

Cheung, V. G., Nayak, R. R., Wang, I. X., Elwyn, S., Morley, S. M. C. M., and Spielman, R. S. (2010). Polymorphic cis- and trans-regulation of human gene expression. *PLoS Biology*, 8:14.

Frazee, A. C., Langmead, B., and Leek, J. T. (2011). Recount: a multi-experiment resource of analysis-ready rna-seq gene count datasets. *BMC Bioinformatics*, 12:449.

Montgomery, S. B., Sammeth, M., Gutierrez-Arcelus, M., Lach, R. P., Ingle, C., Nisbett, J., Guigo, R., and Dermitzakis, E. T. (2010). Transcriptome genetics using second generation sequencing in a caucasian population. *Nature*, 464:773–777.

Pickrell, J. K., Marioni, J. C., Pai, A. A., Degner, J. F., Engelhardt, B. E., Nkadori, E., Veyrieras, J.-B., Stephens, M., Gilad, Y., and Pritchard, J. K. (2010). Understanding mechanisms underlying human gene expression variation with rna sequencing. *Nature*, 464:768–772.
